# New Perspectives on Genetic Prediction for Pediatric Metabolic Associated Fatty Liver Disease

**DOI:** 10.3389/fped.2020.603654

**Published:** 2020-12-09

**Authors:** Yu-Cheng Lin, Chi-Chien Wu, Yen-Hsuan Ni

**Affiliations:** ^1^Department of Pediatrics, Far Eastern Memorial Hospital, New Taipei City, Taiwan; ^2^Department of Healthcare Administration, Oriental Institute of Technology, New Taipei City, Taiwan; ^3^Departments of Pediatrics, National Taiwan University Hospital, Taipei, Taiwan

**Keywords:** fatty liver, genetics, sequence variation, precision medicine, pediatric, children, obesity

## Abstract

Non-alcoholic or recently re-defined metabolic associated fatty liver disease (MAFLD), a spectrum of progressive hepatic disease, has become a public health issue in obese children and adolescents. MAFLD is a complex metabolic disease strongly associated with obesity and insulin resistance. It is not known why not every obese subject will develop MAFLD. Different ethnic/racial groups display differences in MAFLD prevalence, indicating genetic factor plays a role. In the past two decades, sequence variations in genetic loci, including *PNPLA3, TM6SF2, GCKR, MBOAT7, HSD17B13*, etc. have been shown to confer susceptibility to MAFLD in children and adults. This review article provides an updated viewpoint of genetic predictors related to pediatric MAFLD. We discuss whether these susceptible genes can be clinically used for risk stratification and personalized care. Understanding human genetics and molecular mechanisms can give important information not only for prediction of risk but also on how to design drugs. In view of current epidemic of MAFLD worldwide, it is necessary to identify which children with MAFLD progress rapidly and need earlier intervention. In the future, a comprehensive analysis of individualized genetic and environmental factors may help assess the risk of children with MAFLD and personalize their treatment.

## Introduction

There is increasing interest in metabolic associated fatty liver disease (MAFLD), defined as excessive deposition of fat in the liver in the absence of significant alcohol consumption. The progression of MAFLD encompasses a spectrum of conditions ranging from fat in the liver—simple steatosis, fat with inflammation and/or fibrosis-steatohepatitis to advanced fibrosis and cirrhosis over time (1). MAFLD is one of the most common chronic liver disease in the whole world (2, 3), becoming a global health burden (4). The long-term follow-up study revealed adults with MAFLD had increased liver related and non-liver related mortalities (5). MAFLD is now a serious health condition not only for adults, but also for children (6).

Pathogenesis of MAFLD is complicated, multifactorial (7), and strongly associated with obesity related comorbidities such as insulin resistance, cardiac dysfunction, and kidney disease, etc. (8–11). Nowadays, MAFLD is no longer regarded as a primary hepatic disease, but rather a component of metabolic syndrome. Therefore, a recent expert consensus group suggested the metabolic associated fatty liver disease “MAFLD” as a more appropriate term than the nomenclature of non-alcoholic fatty liver disease (NAFLD). Since children drink less alcohol, we believe that the term MAFLD is more suitable for children and should replace NAFLD (12).

Due to a continuum from obesity to metabolic syndrome, patients with MAFLD may benefit from early identification of the disease risk and individually targeted treatment (13). Establishing clinical predictors is necessary for MAFLD diagnosis and risk stratification. Previously reported biochemical factors include elevated total cholesterol, triglycerides, fasting insulin, increased fasting glucose and insulin concentrations, homeostatic model assessment for insulin resistance (HOMA-IR) index, and aspartate aminotransferase (AST)/alanine aminotransferase (ALT) ratio (14, 15).

There are several differences between pediatric and adult MAFLD in prevalence, risk propensity, and liver histology (Table 1) (20, 21). Risk factors such as alcohol abuse, drug abuse and comorbidities among children are much less than those in adults. Understanding the differences between pediatric and adult MAFLD can help assess how NAFLD progresses from childhood toward adulthood. The histological pattern of pediatric MAFLD is different from that of adults. The classic histological findings that represent MAFLD are: steatosis, swelling, inflammation, and fibrosis. In adults, steatosis, inflammation, and accumulation of collagen start in the perivenular area (zone 3), while in children, it usually starts in the periportal area (zone 1) with lack of ballooning (22). However, it is not clear whether patients with pediatric MAFLD pattern differ in pathogenesis, prognosis, or response to treatment (23). In addition, due to lack of long-term follow-up from children to adults, the natural history and prognosis of MAFLD in children are still uncertain. Compared with adults with MAFLD, children with MAFLD have a much longer course of disease. Reversing the course of MAFLD in childhood is indeed an unmet need.

**Table 1:** Differences between adult and pediatric MAFLD.

**Clinical Features**	**Children**	**Adults**
Prevalence (4, 16)	7.6% overall, and 34.2% obese	24% overall, and 45–70% obese
Histology (17)		
Steatosis	Typically zone 1	Typically zone 3
Inflammation	Mainly portal	Mainly lobular
Ballooning	Rale	Common
Fibrosis	Predominantly portal-periportal	Perisinusoidal chicken wire
Cirrhosis (18)	1–2%	5–10%
Prognosis marker (19)	Unknown (lack of long-term longitudinal studies)	Degree of fibrosis

## Epidemiology

The increasing epidemic of obesity and sedentary lifestyle continues to raise the prevalence of MAFLD (24). As the global obesity epidemic worsens metabolic disorders, the health burden of children's MAFLD has become huge (16, 25). Recent research indicated in obese children, MAFLD is present in nearly one-third of boys and one-fourth of girls (26). Not every obese subject will suffer from MAFLD, which suggests that genetic and/or environmental factors contribute to each individual's susceptibility. In fact, MAFLD can occur in non-obese individuals (27, 28).

Different ethnic/racial groups display differences in MAFLD prevalence (29). It is recognized the highest prevalence is in the American Hispanic population followed by the Caucasian and the African-American (30). In pediatric population, obese Hispanic adolescents are more likely to develop MAFLD than obese non-Hispanic adolescents (31). Compared with the West, the East has a lower MAFLD incidence and prevalence (32).

Different lifestyle and nutrition status may partially account for the differences among ethnic groups. However, the Western diet and sedentary lifestyle have led to the emergence of obesity and MAFLD in Asia over the last decade (33). On the other hand, growing evidence reveals the importance of genetic factors in the development and progression of MAFLD. Since the frequency of genetic variants differs among ethnic groups, increasing understanding of the genetic predisposition to MAFLD may help us to decipher the reasons for its occurrence (34).

## Heritability of MAFLD

There is growing awareness of the role of genetic factors in the etiology and prognosis of MAFLD. The differences in disease distribution observed in adults and children with MAFLD indicate genetic susceptibility plays a crucial role in the development of MAFLD (6). In a twin study, Loomba et al. revealed that MAFLD-associated hepatic steatosis and fibrosis are heritable traits (35). Genome-wide association studies (GWAS) have identified several important single nucleotide polymorphisms (SNPs) affecting the severity and progression of MAFLD (36). Overall, the dynamic interactions between genetic and environmental factors further modulate the disease phenotype, susceptibility, development, and progression (37–39).

Till now, MAFLD susceptible genes have been reported to be involved in a wide spectrum of pathogenic mechanisms, including lipid metabolism, insulin signaling, oxidative stress, inflammation and fibrogenesis, etc. Genetic factors do not have the same effects across studies due to different study populations and designs. For example, people with *PNPLA3* variant are usually considered to have more hepatic steatosis, inflammation, and fibrosis. However, Kotronen et al. reported *PNPLA3* SNP did not improve the prediction of liver fat content by using their liver fat score equation (40). In fact, MAFLD is a polygenetic disease and we need more genetic information, rather than just one SNP, to establish the predictive model.

## Major Common Genetic Variations of MAFLD

Herein, we provide a comprehensive update on genetic variations related to pediatric MAFLD (Table 2). The genetic determinants of MAFLD not only can predict the progression of MAFLD, but also are the possible targets for therapy.

**Table 2:** Genetic variations associated with pediatric MAFLD.

**Gene**	**Variant**	**Chr**.	**Population (ethnicity)**	**Subjects (n)**	**Function**	**Consequence**	**Phenotype**	**References**
*PNPLA3*	rs738409 C>G	22q13.31	Mexican	1,037	Lipid droplets remodeling	Ile148Met	↑MAFLD, Fibrosis	(41)
			Taiwanese	520				(42)
			Hispanic	327				(43)
			Caucasian	149				(44)
*TM6SF2*	rs58542926 C>T	19p13.11	Italian	1,010	Modulate hepatic VLDL secretion	Glu167Lys	↑MAFLD, Fibrosis	(45)
			402 Caucasians 266 African Americans 289 Hispanics	957				(46)
			Taiwanese	831				(47)
GCKR^*^	rs780094 C>T	2p23	Taiwanese	797	Modulate hepatic lipogenesis	Intronic variant	↑MAFLD, Fibrosis	(48)
	rs1260326 C>T		181 Caucasians 139 African Americans 135 Hispanics	455		Leu446Pro		(49)
MBOAT7^**^	rs641738 C>T	19q12.42	Italian	1,002	Remodeling of phosphatidylinositol	Glu17Val	↑MAFLD, Fibrosis	(50)
			Caucasian	467				(51)
			Taiwanese	831			No effect	(47)
	rs626283 G>C		Caucasian	860		Intron variant	↑MAFLD	(52)
*HSD17B13*	rs72613567: TA	4q22.1	Italian	685	Retinol dehydrogenase activity	Splice donor variant	↓MAFLD, Fibrosis	(53)
*IRGM*	rs10065172 C>T	5q33.1	Taiwanese	832	Alter hepatic lipophagy	Leu105=	↑MAFLD	(54)
			Italian	613				(55)
*MTTP*	rs2306986 G>C	4q23	Han Chinese	368	VLDL secretion	Glu98Asp	↑MAFLD, Fibrosis	(56)
*LPIN1*	rs13412852 C>T	2p25.1	Italian	142	Lipogenesis	Intronic variant	↑MAFLD, Fibrosis	(57)
*IRS-1*	rs1801278 A>G	2q36.3	Italian	71	Impair insulin signaling	Gly971Arg	↑Fibrosis	(58)
*ENPP1*	rs1044498 A>C	6q23.2	Italian	71	Inhibit insulin signaling	Lys173Glu	↑MAFLD, Fibrosis	(58)
			German	70				(59)
*GPR120*	rs116454156 G>A	10q23.33	Italian	581	Modulate inflammation response	Arg270His	↑MAFLD	(60)
*UGT1A1*	rs4148323 G>A	2q37.1	Taiwanese	234	Increase bilirubin with anti-oxidant activity	Gly71Arg	↓MAFLD	(61)
*PPARGC1A*	rs8192678 G>A	4p15.1	Taiwanese	781	Regulate cellular energy metabolism	Gly487Ser	↑MAFLD	(62)
*HO-1*	(GT)n repeat	22q12	Taiwanese	101	Anti-oxidative stress	Promoter activity	↑MAFLD	(63)
*CNR2*	rs35761398 A>G	1p36.11	Italian	118	Modulate inflammation response	Gln63Arg	↑MAFLD	(64)
*KLB*	rs17618244 G>A	4p14	Italian	249	Upregulate lipotoxic and proinflammatory genes	Arg728Gln	↑MAFLD	(65)
*KLF6*	rs3750861 G>A	10p15.2	Italian	152	Regulate hepatic stellate cell activation and fibrogenesis,	IVS1-27A	↓Fibrosis	(66)
*FDFT1*	rs2645424 A>G	8p23.1	87 Caucasians 61 African Americans 81 Hispanics	229	Modulate intrahepatic cholesterol biosynthesis	Intronic variant	↑MAFLD	(67)

Chr, chromosome; MAFLD, metabolic associated fatty liver disease; VLDL, very low-density lipoprotein.

* GCKR rs780094 and rs1260326 are in strong linkage disequilibrium.

** *MBOAT7 rs641738 and rs626283 are in strong linkage disequilibrium*.

To date, several major common MAFLD susceptible genes reported in adults have been replicated in pediatric studies, such as *PNPLA3, TM6SF2, GCKR, MBOAT7*, and *HSD17B13*. The effects and presumptive functions of these susceptible genes are discussed as follows:

### PNPLA3

Patatin-like phospholipase domain containing 3 (*PNPLA3*) gene encodes a transmembrane protein “adiponutrin,” which is expressed predominantly in the liver, retina, skin, and adipose tissue (68, 69). In 2008, Romeo et al. first identified the association of a *PNPLA3* gene I148M variant with hepatic fat content in adults by GWAS (70). Subsequently, Romeo et al. reported this variant was associated with increased levels of ALT/AST in obese children, which suggests that it confers a genetic susceptibility to liver damage since childhood (71). Furthermore, *PNPLA3* gene I148M variant modulates the progression and liver-related outcomes in patients with MAFLD (72). The effects of *PNPLA3* variants on pediatric MAFLD have been validated in different ethnicities, including Han Chinese, Hispanic, and Caucasian (41–44). Till now, *PNPLA3* is regarded as the most robust susceptible gene for MAFLD across different ethnicities.

The wild type PNPLA3 protein facilitates triglyceride hydrolysis. Impaired hydrolysis of triglycerides/lipid droplet (LD) remodeling in hepatocytes leads to hepatic steatosis (73, 74). PNPLA3 I148M promotes the accumulation of intracellular lipids in the liver by reducing the lipidation and secretion of low-density lipoprotein (VLDL) particles (75). Further studies showed the *PNPLA3* variant not only increases the odds of developing fatty liver itself, but it also determines the degree of hepatic injury and the full spectrum of histopathologic consequences of MAFLD (76). Several studies have shown that PNPLA3 increases the stimulation of hepatic stellate cells by affecting the metabolism of retinoids, leading to liver fibrosis (77–79). Hence, *PNPLA3* SNP has emerged as the key genetic determinant of MAFLD severity in both adults and pediatric patients (80). A recent study showed that silencing *Pnpla3* with antisense oligonucleotides improved liver steatosis and fibrosis in *Pnpla3* I148M knock-in mice, indicating that PNPAL3 could be a potential target for treatment in human (81).

In addition to the effect on liver, *PNPLA3* rs738409 variant has been reported to be associate with reduced glomerular filtration rate (GFR) in children with obesity, indicating the variant *PNPLA3* genotype may be related to kidney dysfunction in children independent of MAFLD status (82, 83).

### TM6SF2

The human transmembrane 6 superfamily member 2 (*TM6SF2*) gene encodes a protein of 351 amino acids with 7–10 predicted transmembrane domains. TM6SF2 protein facilitates the transfer of neutral lipids from cytoplasmic to luminal LDs and VLDL particles. Overexpression of *TM6SF2* decreases the number and size of LDs (84).

In 2014, Kozlitina et al. reported that the *TM6SF2* rs58542926 variant, a C-to-T substitution, encoding a glutamate to lysine change at codon 167 (E167K) and associated with high hepatic triglyceride content and elevated liver serum enzymes levels in adults enrolled in the Dallas Heart Study (85). *TM6SF2* E167K variant carriers with MAFLD have impaired hepatic lipid synthesis from polyunsaturated fatty acids (86). Further studies reported the *TM6SF2* rs58542926 variant also influences hepatic fibrosis and metabolic homeostasis (87, 88). In children, the association between *TM6SF2* rs58542926 variant and MAFLD has been replicated in different ethnicities (45–47). Interestingly, the plasma levels of triglycerides are lower in *TM6SF2* E167K variant carriers than in the non-carriers (89). The increase in hepatic steatosis for loss of function mutation in *TM6SF2* is due to a double mechanism, namely a reduction in the lipidation of VLDL particles (84) and in the number of the secreted apolipoprotein B100 particles (90). In addition to its effects on the liver, it has been reported that *TM6SF2* variants also affected the renal function in children (91) and adults (92) independently of MAFLD.

### GCKR

Glucokinase regulator protein (GCKR) is an inhibitor of glucokinase which regulates glucose storage and disposal and controls *de novo* lipogenesis by regulating the flux of glucose into hepatocytes (93). In 2011, Speliotes et al. reported variants in or near *GCKR* are associated with liver fat content and histopathologic phenotypes at genome-wide significance levels (94). Subsequent meta-analysis provides evidence of significant association between *GCKR* rs780094 (an intronic variant) and risk of MAFLD (95). Silva et al. measured metabolites by mass spectrometry and found novel associations of the *GCKR* rs780094 variant with amino acids and their downstream metabolites, especially lipids (96). In addition, the progression of fibrosis in MAFLD could be influenced by the *GCKR* genotype (97). The effect of *GCKR* genotype on pediatric MAFLD have been reported. The variant *GCKR* rs780094 has been reported to confer susceptibility to MAFLD in obese school children and adolescents in Taiwan (48).

Another common variant in *GCKR* rs1260326, which is in linkage disequilibrium with rs780094, was associated with hepatic triglyceride content in the Dallas Heart Study (98). This variant encodes for a proline to leucine substitution at the 446 position (P446L), resulting in a loss of the affinity of the GCKR protein for the glucokinase. Consequently, more glucokinase is available in the cytoplasm to convert glucose into glucose-6-phosphate. The increased production of glucose-6-phosphate results in an increased rate of glycolysis, leading to increased production of malonyl-CoA, the precursor of *de novo* lipogenesis (99). The rs1260326 in *GCKR* gene is also linked to fatty liver in obese youths (49).

### MBOAT7

The membrane-bound O-acyltransferase domain-containing protein 7 (*MBOAT7*) is a 6 transmembrane domain (100). Hepatocyte specific inactivation of this gene caused an increase in hepatic fat content due to a non-canonical triglyceride synthesis pathway related to a high turnover of phosphatidyl inositol (101). Down-regulation of *MBOAT7* predisposes subjects to MAFLD (102).

In 2016, Mancina et al. first reported the rs641738 C>T variant in the *MBOAT7* gene was associated with increased risk of MAFLD in adults of European descent (103). Subsequently, an association between *MBOAT7* variant and liver fibrosis severity was confirmed by Krawczyk et al. (104). In an animal study, *MBOAT7* over-expression was negatively correlated with obesity and insulin sensitive, driving the progression of MAFLD (105). A pediatric study showed the *MBOAT7* rs641738 variant was associated with plasma concentrations of ALT in obese children (50, 51). Umano et al. reported that another variant (rs626283) in *MBOAT7* gene was associated with MAFLD in Caucasian obese children (52). Notably, conflicting data has reported that *MBOAT7* rs641738 polymorphism does not influence hepatic steatosis and liver injury as determined by serum levels of CK-18 fragment in obese Taiwanese children of Han Chinese ethnicity (47). The difference in the effect of *MBOAT7* variant on MAFLD might be due to different ethnicities.

### HSD17B13

Increasing data demonstrate hydroxysteroid 17-beta dehydrogenase 13 (HSD17B13) acts a pivotal part in hepatic lipid homeostasis and the pathogenesis of MAFLD (106). In 2018, Abul-Husn et al. reported that a loss-of function splice variant (rs72613567:TA) of *HSD17B13* gene was associated with a reduced risk of chronic liver disease and of MAFLD progression (107). More recently, an exome-wide association study confirmed the *HSD17B13* rs72613567 variant influenced the susceptibility and histological severity of MAFLD (108). A pediatric study in Italy, reported obese children carrying the *HSD17B13* variant had lower hepatic steatosis and pediatric MAFLD fibrosis index than non-carriers (53). A recent work also showed that this genetic variation provided kidney protection for children (109).

The HSD17B13 protein is a liver-specific LD-associated protein and exhibits retinol dehydrogenase activity. The *HSD17B13* variant is supposed to alter mRNA splicing, yielding a truncated protein with reduced enzymatic activity. However, the exact role and function of HSD17B13 in MAFLD pathophysiology remains largely uncharacterized. Over-expression of *HSD17B13* in human hepatoma cell lines or C57BL/6 mice leads to excessive lipid accumulation. *HSD17B13* gene encodes a LD-associated protein, which is involved in regulating lipogenesis (110). Recently, the Rotman group published interesting studies on the inactivation of *Hsd17b13* in mice (111) and the identification of an enzymatic active site metabolizing retinol (112).

## Hepatic Lipid Metabolism

Excessive fat accumulation damages hepatocytes and leads to inflammatory response, cytokine production, oxidative stress, abnormal cellular signaling, and activation of stellate cells. The intracellular storage and utilization of lipids play an important role in supporting cellular energy homeostasis. In addition to the major MAFLD susceptible genes aforementioned, several important genetic variants have been identified to affect hepatic lipid metabolism.

### IRGM

In 2009, Singh et al. first reported that LDs can be degraded in hepatocytes by a specific autophagy-related process “lipophagy” (113). The immunity-related GTPase family M protein encoded by the *IRGM* gene controls autophagy activation (114). Our previous study found obese children with the variant *IRGM* rs10065172 TT genotype have a higher risk of MAFLD and elevated ALT levels compared with subjects with wild type (54). A downregulation of *IRGM* in HepG2 cells decreased autophagic flux accompanied by an increased lipid accumulation. In contrast, overexpression of *IRGM* decreased LD content in HepG2 cells. This genetic association has been replicated in a cohort of obese Italian children (55). A recent murine study revealed liver specific suppression of *Ifgga2* (the mice ortholog of human *IRGM*) increases hepatic fat content in a backcross of obese C57BL/6J New Zealand mice (115).

### MTTP

The human microsomal triglyceride transfer protein (MTTP) works to lipidate and assemble the apoB-containing lipoproteins in the liver. It is critical to remove lipid from liver through the assembly and secretion of VLDL particles. Hsiao et al. reported the *MTTP* polymorphisms can modulate lipid homeostasis and determine the serum lipids and risk of MAFLD (116). A pediatric study also showed the association of *MTTP* rs2306986 variant and MAFLD in obese children (56).

### LIPIN1

Lipin-1 encoded by the *LPIN1* gene, expressed mainly in adipose and the liver, has phosphatidic phosphatase activity (117). Valenti et al. reported children, but not adult, carrying the *LIPIN1* rs13412852 TT genotype had a lower prevalence of MAFLD, less severe liver damage and a lower liver fibrosis prevalence (57). The mutation in *LIPIN1* gene may result in decreasing the flux of free fatty acids (FFAs) to the liver.

## Insulin Resistance

Insulin resistance is a feature of the MAFLD pathophysiology and occurs in its early phases (118, 119). It affects metabolic syndrome and MAFLD in obese children and adolescents (120), even in non-obese patients (121). Peripheral insulin resistance leads to excessive lipolysis in adipose tissue, releasing a lot of FFAs into the circulation (8). The liver then uptakes excessive FFAs and exceeds its capacity to transfer FFAs into neutral triglycerides, causing hepatic steatosis, lipotoxicity, and endoplasmic reticulum stress (122).

### IRS1, ENPP1

The insulin receptor substrate 1 (*IRS1*) and ectonucleotide pyrophosphate phosphodiesterase (*ENPP1*) genes play crucial roles in controlling cell signaling in response to insulin. Once insulin binds to the insulin receptor, the IRS1 protein regulates hepatic gene expression that coordinates glucose homeostasis. ENPP1 protein negatively modulates insulin receptors and induces insulin resistance if overexpressed.

Hepatic *IRS1* overexpression is associated with histological progression in patients with MAFLD (123). The *ENPP1* rs104449 and *IRS1* rs1801278 variants decrease hepatic insulin signaling and predispose adult patients with MAFLD to liver damage (58). In children, Hudert et al. reported *ENPP1* rs1044498, not *IRS1* rs1801278 variant was associated with pediatric MAFLD (59).

## Oxidative Stress and Inflammation

The two-hit hypothesis is widely recognized as a model of MAFLD progression (124). The first hit causes hepatic fat accumulation and the second hit causes inflammation and fibrosis. The second hit usually results from excessive oxidative stress, such as mitochondrial stress and insulin resistance. In the other words, oxidative stress plays a critical role in the progression from simple steatosis to steatohepatitis (125, 126).

### GPR120

G protein-coupled receptor 120 (GPR120) is a functional omega-3 fatty acid receptor that mediates anti-inflammatory and insulin sensitivity (127, 128). In 2014, Marzuillo et al. reported the association between the *GPR120* rs116454156 variant (R270H) and liver injury in obese children and adolescents (60). By regulating GPR120, docosahexaenoic acid (DHA) can reduce the inflammatory response of MAFLD in children (129).

### UGT1A1

Genetic modifiers belonging to oxidative stress are involved in either the generation of reactive oxygen species (ROS) or the modulation of cellular antioxidant defense. Genetic variant in uridine-5'-diphosphoglucuronosyltransferase 1A1 (*UGT1A1*) increases serum bilirubin, which has anti-oxidative properties. Our previous study found that the variant UGT1A1^*^6 genotype was associated with a lower risk of MAFLD in obese Taiwanese children (61). A subsequent study showed an inverse relation between serum bilirubin levels and the presence of MAFLD on Italian children, replicating our previous finding (130).

### HO-1

Increasing heme oxygenase-1 (HO-1) activity can reverse complications related to obesity, metabolic syndrome, and MAFLD (131). HO-1 is a stress-responsive protein, defensing against the oxidative process. The (GT)n dinucleotide repeat within the *HO-1* gene promotor region is highly polymorphic. Our previous study found that obese children with the long repeat of *HO-1* (GT)n dinucleotide were more susceptible to MAFLD (63). In obese mice, liraglutide, a glucagon-like peptide 1 analog, ameliorated MAFLD severity through upregulating Sestrin2-mediated Nrf2/HO-1 pathway in obese mice (132), suggesting HO-1 could be a therapeutic target for MAFLD.

### PPARGC1A

The peroxisome proliferator-activated receptor gamma coactivator 1-alpha (*PPARGC1A*) gene encodes a PGC-1α protein that regulates mitochondrial functions, oxidative stress, and lipogenesis (133). Our previous study revealed the *PPARGC1A* rs8192678 risk A allele was associated with an increased risk of MAFLD in obese Taiwanese children (62). An animal study revealed loss of estrogen signaling contributes to oxidative damage caused by low levels of PPARGC1A in liver in mice (134).

### CNR2

Innate immunity and inflammation are the hallmarks of progressive MAFLD (135). Inflammatory cytokines are involved in the progression from simple steatosis to steatohepatitis. The cannabinoid receptor type 2 (CB2), encoded by the *CNR2* gene, is a seven-transmembrane domain G protein-coupled receptor. Activation of CB2 receptor inhibits nuclear translocation of NF-kB, decreasing production of inflammatory cytokines. An animal experiment demonstrated CB2 activation can reduce hepatic injury and promote liver regeneration (136). Recent research revealed the role of the *CNR2* rs35761398 variant in modulating the hepatic inflammation state in obese children with MAFLD and in increasing susceptibility to liver damage (64).

### KLB

β-Klotho gene (*KLB*) encodes a transmembrane protein mainly expressed in the liver. A recent study indicated *KLB* rs17618244 variant increased the risk of hepatocellular ballooning and lobular inflammation in children with MAFLD (65). The precise mechanism of KLB protein on MAFLD is not clear.

## Fibrogenesis

The liver fibrosis stage is the strongest predictor for disease-specific mortality in MAFLD (137). Accurate assessment of the risk of having advanced liver fibrosis in children with MAFLD is important in the clinical practice. However, because the fibrosis phenotype requires a period of cumulative damage, it is difficult to accurately measure the impact of genetic variation on the liver fibrosis in children with MAFLD. This is why there are fewer studies on the association between liver fibrosis and MAFLD in children. The limited available data on genetic variants associated with liver fibrosis in children with MAFLD are discussed below:

### KLF6

Nobili et al. reported the Krueppel-like factor 6 (*KLF6*) rs3750861 variant reduced the risk of liver fibrosis in children with MAFLD (66). *KLF6* is up-regulated by activated HSCs following liver injury (138).

### FDFT1

The farnesyl-diphosphate farnesyltransferase 1 (*FDFT1*) gene encodes for squalene synthase and modulates the cholesterol biosynthesis. This *FDFT1* rs2645424 variant has been reported to be associated with the MAFLD activity score and moderate/severe fibrosis in a multiethnic cohort of obese youths (67). It is not currently clear whether the *FDFT1* rs2645424 variant affects the enzyme activity because it is an intronic variant.

## Influences of Genetic Variants on MAFLD Pathogenesis

**Figure 1:**
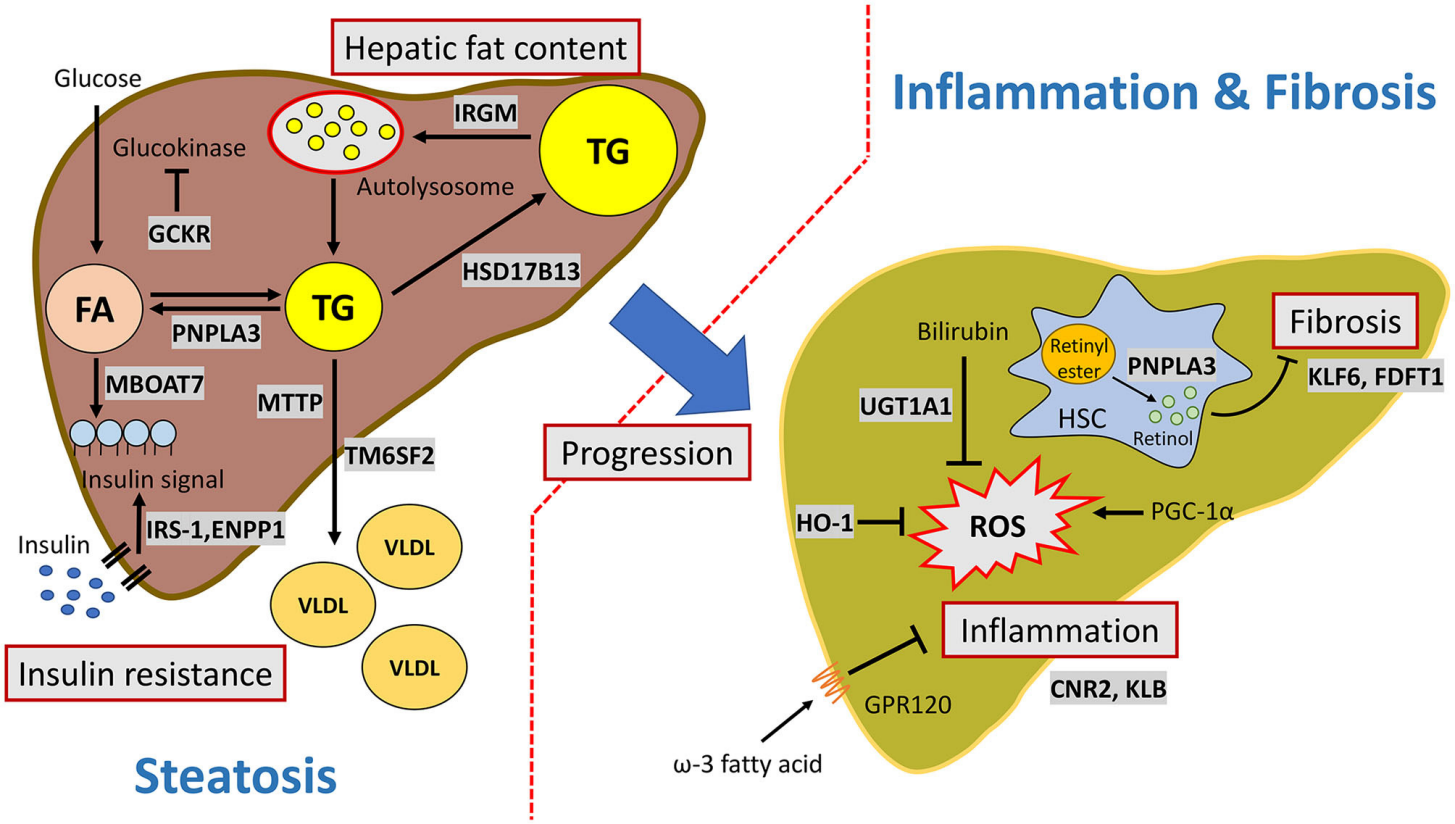
Major pathways involved in the genetic susceptibility toward MAFLD. PNPLA3 facilitates triglyceride hydrolysis in hepatocytes and mediates retinol release from retinyl ester in hepatic stellate cells. IRGM activates autophagosome formation and increases hepatic lipophagy. TM6SF2 transfers triglyceride from cytoplasmic to VLDL particles. MBOAT7 is involved in phospholipid remodeling. HSD17B13 is a lipid droplet-related protein and has retinol dehydrogenase activity. IRS-1 and ENPP1control cell signaling in response to insulin. GCKR regulates glucokinase activity and facilitates *de novo* lipogenesis. GPR120 is a functional omega-3 fatty acid (such as DHA) receptor which mediates anti-inflammatory effects. PGC-1α (encoded by *PPARGC1A*) is a master regulator of reactive oxygen species. HO-1 and UGT1A1are involved in the bilirubin metabolism and antioxidant activity. CB2 (encoded by *CNR2*) and KLB are involved in the inflammation process, but the mechanism is still unknown. KLF6 and squalene synthase (encode by *FDFT1*) are associated with fibrosis. KLF6 activates hepatic stellate cells. Squalene synthase modulates cholesterol biosynthesis.

A schematic representation of the pathways in the pathogenesis of MAFLD development and progression affected by genetic factors is summarized in Figure 1. It currently remains difficult to accurately predict the development and progression of MAFLD because of the complicated interactions between genetic pathways and variable environmental influences.

## Translational Implications of Genetic Variations for MAFLD

Genetic variations in *PNPLA3, TM6SF2, GCKR, MBOAT7*, and *HSD17B13*, etc. provide novel insights into the MAFLD pathophysiology and may be incorporated into predictive model for precision medicine in patients with MAFLD (139). Early recognition and treatment of MAFLD decreases long-term morbidity and mortality (140). Because MAFLD is a heterogeneous disease, the treatment option should be personal/ individualized. Personalized prediction is required to guide risk stratification and treatment. Ma et al. reported improved diet quality is more effective in individuals at a high genetic risk of MAFLD (141). DHA supplementation may not be as effective as non-carriers in reducing liver fat levels in *PNPLA3* I148M carriers (142). Recently, Costanzo et al. reported a weighted-genetic risk score combining *PNPLA3, GCKR*, and *TM6SF2* risk alleles was associated with an 8-fold higher risk of MAFLD in obese children (143). In this regard, polygenic risk scores may be applied in risk stratification and guide the treatment.

Modulation of the human genetics associated with MAFLD presents the opportunity to develop a precision medicine. High-throughput technologies, such as gene array and next generation sequencing, can facilitate the translation of genetic testing in the care of children with MAFLD (144). Considering this complexity, computational models may help design personalized treatment strategies which account for genetic and environmental factors (145).

Despite its promise, the utility of genetic testing in patients with MAFLD remains controversial due to the lack of established evidence related to clinical benefits. Societal guidelines from ESPGHAN (European Society for Pediatric Gastroenterology Hepatology and Nutrition) in 2012 and NASPGHAN (North American Society for Pediatric Gastroenterology, Hepatology, and Nutrition) in 2017 recognize the genetic predisposition strongly affects the risk of MAFLD development in children, but they do not recommend routine genetic testing in children with NALFD (146, 147). In adults, 2016 EASL (European Association for the Study of the Liver) and 2018 AASLD (American Association for the Study of Liver Diseases) guidelines claimed that testing for genetic variants of MAFLD in routine clinical care is currently not advocated (148, 149). More trials are needed to be conducted to test the role of gene-based diagnosis and treatment for MAFLD before its clinical use.

## Conclusion

This review article summarizes current knowledge and new advances related to the genetics of pediatric MAFLD. Overall, understanding human genetics and molecular mechanisms can give important information not only for prediction of risk but also on how to design drugs (150). To date, genetic studies have successfully advanced our understanding in the pathogenesis of MAFLD, but there are still gaps in translating these genetic studies into clinical applications in the real world. In the future, by analyzing more comprehensive personalized genetic and environmental factors, we will be able to accurately assess the risk of children with MAFLD and adopt personalized treatment.

## Author Contributions

All authors listed have made a substantial, direct and intellectual contribution to the work, and approved it for publication.

## Conflict of Interest

The authors declare that the research was conducted in the absence of any commercial or financial relationships that could be construed as a potential conflict of interest.
